# Exploiting mutual information for the imputation of static and dynamic mixed-type clinical data with an adaptive k-nearest neighbours approach

**DOI:** 10.1186/s12911-020-01166-2

**Published:** 2020-08-20

**Authors:** Erica Tavazzi, Sebastian Daberdaku, Rosario Vasta, Andrea Calvo, Adriano Chiò, Barbara Di Camillo

**Affiliations:** 1 Department of Information Engineering, University of Padua, Via Gradenigo 6/A, Padua, 35131 Italy; 2 Department of Neurosciences “Rita Levi Montalcini”, University of Turin, Via Cherasco 15, Turin, 10124 Italy

**Keywords:** Imputation, Missing data, K-nearest neighbours, Mutual information, Naïve Bayes, Clinical datasets, Amyotrophic lateral sclerosis

## Abstract

**Background:**

Clinical registers constitute an invaluable resource in the medical data-driven decision making context. Accurate machine learning and data mining approaches on these data can lead to faster diagnosis, definition of tailored interventions, and improved outcome prediction. A typical issue when implementing such approaches is the almost unavoidable presence of missing values in the collected data. In this work, we propose an imputation algorithm based on a mutual information-weighted k-nearest neighbours approach, able to handle the simultaneous presence of missing information in different types of variables. We developed and validated the method on a clinical register, constituted by the information collected over subsequent screening visits of a cohort of patients affected by amyotrophic lateral sclerosis.

**Methods:**

For each subject with missing data to be imputed, we create a feature vector constituted by the information collected over his/her first three months of visits. This vector is used as sample in a k-nearest neighbours procedure, in order to select, among the other patients, the ones with the most similar temporal evolution of the disease over time. An ad hoc similarity metric was implemented for the sample comparison, capable of handling the different nature of the data, the presence of multiple missing values and include the cross-information among features captured by the mutual information statistic.

**Results:**

We validated the proposed imputation method on an independent test set, comparing its performance with those of three state-of-the-art competitors, resulting in better performance. We further assessed the validity of our algorithm by comparing the performance of a survival classifier built on the data imputed with our method versus the one built on the data imputed with the best-performing competitor.

**Conclusions:**

Imputation of missing data is a crucial –and often mandatory– step when working with real-world datasets. The algorithm proposed in this work could effectively impute an amyotrophic lateral sclerosis clinical dataset, by handling the temporal and the mixed-type nature of the data and by exploiting the cross-information among features. We also showed how the imputation quality can affect a machine learning task.

## Background

By discovering novel and useful patterns from clinical registers and electronic health records, healthcare analytics has transformed the healthcare industry both in terms of cost optimisation and ever improving quality of care [1]. Among the possible approaches, the use of machine learning (ML) and data mining techniques are providing the means to extract information from the complex and voluminous amount of available data, virtually creating a paradigm shift in the whole healthcare sector, from basic research to clinical and management applications [2, 3]. The possible advantages of such analyses could vastly improve patients’ lives and benefit society as a while. From an economic perspective, the use of these techniques to improve practice efficiency results in a more affordable, high-quality healthcare [4]. Besides, from a clinical point of view, the possible improvements in medical knowledge, as well in diagnosis and prognosis capabilities, allow higher health standards. Studies as survival analyses can evidence risk factors and detect the effect of specific treatments both in disease progression and quality of life [5], moving towards a personalised care system. Moreover, an enhanced knowledge of the pathologies can be translated into computer-aided tools, offering clinicians a valid support in decision making.

The creation of accurate and effective analytic models from healthcare data, however, is challenging, because of issues regarding quality and heterogeneity [6]. The type and frequency of collected data vary based on the specific application field, a patient’s clinical condition and administrative requirements. Moreover, medical tests and treatments can be carried out at different times even if patients exhibit the same symptoms. This, together with human factors (poor handwriting, missing charts or pages, measurements being documented in inconsistent locations, etc.), results in many aspects of a patient’s clinical condition being unmeasured or unrecorded at different time points.

Missing values may be clinically important, but cannot be handled by most analytics algorithms [7] and can significantly affect the conclusions that can be drawn from the data [8]. For instance, missing data can introduce bias in the results of randomised controlled trials, negatively affecting the derived clinical decisions and ultimately patient care [9]. When performing survival analysis, missing data can occur in one or more risk factors. The standard response of simply excluding the affected individuals from the analysis could lead to invalid results if the excluded group is selective with respect to the entire sample, and to a waste of costly collected data [10]. In remote health monitoring settings, missing data is a prevalent issue affecting long-term monitoring systems which can lead to failure in decision making [11]. For electronic health records, missing values frequently outnumber observed ones, mainly because they were designed to record and improve patient care and streamline billing rather than collecting data for research purposes [12].

Many kinds of analyses, from simple statistics to advanced data mining and machine learning methods, either fail altogether in dealing with missing data or end up producing biased estimates of the investigated associations when simple curing techniques (such as complete case analysis, overall mean imputation, or the missing-indicator methods) are applied [13]. To utilise all clinical data and achieve optimal performance of the used algorithms, the missing data issue must be addressed by imputing the missing values.

When considering the heterogeneity of the data recorded in this setting, a typical example of mixed-type variables dataset is represented by disease registers. The variables in this domain can be classified as either *static* if constant throughout the patient’s clinical history, such as sex or age at disease onset, or *dynamic* if varying in time, such as blood pressure or sugar levels at subsequent visits. Furthermore, they can be *continuous* when representing measurements in a range of continuous values, *ordinal* when the values fall in a discrete ordered set, or *categorical* when describing a qualitative property out of a finite number of categories or distinct groups without any order relations. An adequate imputation method should therefore be able to handle this data complexity altogether.

Many of the available imputation methods are restricted to only one type of variable. For mixed-type data, the different variable types are usually handled separately, thus ignoring possible relations among variables of different types. Moreover, most of them make strong assumptions on the characteristics of the missing data, such as locality in Gaussian Process based models [14], low-rankness and temporal regularity in matrix factorisation models [15] and multivariate normality in Expectation-Maximisation methods [16]. Finally, most commonly used imputation methods are not able to explicitly handle the temporal nature of longitudinal patient data [17].

This paper presents an adaptive mutual information-weighted k-nearest neighbours (wk-NN) imputation algorithm developed to explicitly handle missing values of continuous/ordinal/categorical and static/dynamic features conjointly. The proposed methodology was applied and validated on a subset of the Piemonte and Valle d’Aosta Amyotrophic Lateral Sclerosis (PARALS) register [18], a prospective epidemiological register from two Italian regions.

### Types of missing data

Missing values can be of three general types: *missing completely at random* (MCAR), *missing at random* (MAR) and *missing not at random* (MNAR). When missing data are MCAR, the presence and/or absence of data is completely independent of observable variables and parameters of interest. In this case, the set of subjects with no missing data is also a random sample from the source population. This represents the best possible type of missing data as any analysis performed will be unbiased [19], although it is a highly unlikely scenario.

Missing data are MAR when the propensity for a value to be missing depends on some observed patient characteristic. For instance, males are less likely to fill in a depression survey. This kind of missing data can induce bias in the resulting analysis especially when the data is unbalanced because of many missing values in a certain category.

Finally, we are in the MNAR scenario when the missing values are neither MCAR nor MAR. For instance, when asking subjects for their income level it might well be that missing data are more likely to occur when the income level is relatively high. Here, the reason for missingness obviously is not completely at random, but is related to unobserved patient characteristics.

Many imputation methods require the missing data to be MCAR, or at least MAR. On the other hand, an imputation based on a k-nearest neighbours approach is applicable in any of the three previous situations, as long as there is a relationship between the variable with the missing value and the other variables [20].

### Previous work

Several methods for handling missing data are already available [21]. The simplest approaches consists in focusing the analysis only on non-missing values in the dataset, by either dropping cases where at least one variable is missing or by dropping variables where at least one value is missing. These approaches completely neglect the relationships among variables, possibly causing severe information loss and worsening the statistical power and standard errors of the analyses [22, 23]. Mean/median/mode imputation or value propagation (Last Observation Carried Backward or Next Observation Carried Forward), are some other fast and easily interpretable statistical approaches. These imputation methods, however, may lead to low accuracy and biased estimates of the investigated associations [13, 24].

Regression represents a somewhat more advanced imputation approach that estimates missing values by regressing them from other related variables [25], especially time [26]. While deterministic regression limits the imputation to the exact prediction of the regression model, often producing an overestimation of the correlation among the variables, stochastic regression adds a random error term to the predicted value in order to recover a part of the data variability [27].

Multivariate imputation by chained equations (MICE) [28] is one of the most prominent methods in the literature [29]. In this imputation procedure, a series of regression models are run whereby each variable with missing data is modelled conditional upon the other variables in the data. This means that each variable is modelled according to its distribution, with, for example, predictive mean matching for continuous data, logistic regression for binary data, polytomous logistic regression for categorical data and proportional odds for ordinal data.

3D-MICE, recently introduced in [17], combines MICE with Gaussian process (GP) [14, 30] predictions, thus imputing missing data based on both cross-sectional and longitudinal patient data information. MICE is used to carry out cross-sectional imputation of the missing values, while a single-task GP is used to perform longitudinal imputation. The estimates obtained by the two methods are then combined by computing a variance-informed weighted average. 3D-MICE can adequately impute continuous longitudinal patient data, but is unable to handle categorical and static variables.

A non-parametric method based on a random forest that can cope with different types of variables simultaneously, called missForest, was introduced by Stekhoven et al. [31]. This method is based on the idea that a random forest intrinsically constitutes a multiple imputation scheme by averaging over many unpruned classification or regression trees. While not requiring assumptions about distributional aspects of the data, missForest requires the observations to be pairwise independent, which is rarely the case when handling clinical records (several visits for each patient).

Another popular imputation method for cross-sectional time series data is Amelia II [16], which performs multiple imputation by implementing an Expectation-Maximisation with Bootstrapping algorithm. Amelia II can utilise both time series and multi-variable information in a dataset for the imputation task. This method requires all variables in the dataset to be multivariate normally (MVN) distributed. This requirement reduces the applicability of the method especially when dealing with non-normalisable and/or categorical variables.

Recently, a number of deep learning frameworks for estimating missing values in multi-time-series clinical data have been proposed [32–34]. These methods achieved impressive results on benchmark datasets due to the high-quality representations extracted from large amount data, which means that their applicability is limited when only few data are available.

The “nearest neighbours” (NN) methods are among the most popular imputation procedures [20, 35]. Missing values of samples with missing data are replaced by values extracted from similar other samples with respect to observed characteristics. NN imputation approaches are donor-based methods where the imputed value is either a value that was actually measured for another record in a database (1-NN) or the average/median/mode of measured values from *k* records (k-NN). These methods were often shown to outperform other imputation techniques [36], even though results depend heavily on the choice of the metric used to measure the similarity between samples. Moreover, because data collection periods vary across patients, samples may not be directly comparable. Furthermore, the similarity metric should also handle the presence of missing values in the donor samples, manage the different nature of the data, and take into account the possibly unbalanced contribution of static and dynamic variables, with the latter adding information over time.

### Aim of this work

In this work, we present an imputation algorithm based on a weighted k-NN approach, able to handle missing data in static and dynamic mixed-type variables simultaneously. The k-NN imputation approach is fully non-parametric and does not require explicit models to relate variables, thus being less prone to model misspecification than other methods [20]. In our algorithm, we define an ad hoc similarity metric in which we employ the mutual information (MI) values between feature pairs as weights in the computation of the distance among samples, in order to account for the cross-feature information.

The proposed methodology has been developed and validated on a clinical epidemiological register of patients affected by amyotrophic lateral sclerosis (ALS), that is, a collection of dynamically acquired data over subsequent screening visits, one visit at a time. Compared to clinical trial datasets, epidemiological registers better characterise the general ALS population, since clinical trial population must fit a stringent set of criteria [37]. This clinical register represents a typical instance of complex dataset constituted of both static/dynamic and mixed-type variables, and, coherently with its real-world nature, is inevitably subject to missing data.

ALS is a fatal neurodegenerative disorder characterised by progressive muscle paralysis caused by the degeneration of motor neurons in the brain and spinal cord [38]. The disease is progressive and fatal: the symptoms worsen over time and there are no known effective treatments that can effectively halt or reverse its progression, which will inevitably result in respiratory failure, typically within 4 years form disease onset [39]. The enormous social, medical and human costs imposed on ALS patients, their families and the health systems in general are pushing the scientific community towards the development of computational tools to derive predictions for prognostic counselling, stratification of cohorts for pharmacological trials, and timing of interventions [40–44].

To this purpose, two distinct DREAM Challenges have been organised in the past years [41, 44]. By employing the clinical information of the first three months of patients’ visits from different datasets, the participants were asked to develop algorithms to predict the disease progression and to stratify the patients into meaningful subgroups. The PARALS register used in our work was partially included in the datasets of the second challenge.

ALS is a rare disease: its incidence in Europe and in populations of European descent is 2.6 cases for 100,000 people per year and the prevalence is of 7–9 cases per 100,000 people [45], with ALS rates being mainly unknown in the rest of the world [38]. This implies that the available patients’ data collected in clinical registers is of inestimable importance for furthering the translational research on the disease and that missing values cannot be treated with simple curing techniques. With the aim to build a complete dataset from the PARALS register that can be similarly used for the application and development of ML algorithms, we developed an adaptive weighted k-nearest neighbours algorithm for the imputation of the first three months of screening visits. Our imputation method is based on the assumption that subjects with a similar disease progression over a short period of time share similar feature values and can therefore be cross-exploited to impute missing values.

In addition to adequately characterising the temporal evolution of the disease course [41], the selected time interval is short enough to allow the imputation of subjects with few available visits. Moreover, the information of newly added subjects can be promptly used for the imputation of others. Finally, by focusing on a reduced observation interval, only a relatively small number of visits (and thus a relatively small number of features) is considered. In a k-NN setting, having a small number of features prevents the methods from incurring in the curse of dimensionality: in general, as the number of dimensions (features) increases, the closest distance among samples tends to the average distance and the predictive power of the algorithm decreases [46].

The proposed method was compared to three other state-of-the-art imputation algorithms, namely Amelia II [16], missForest [31] and MICE [28], which are the main representatives of the methods currently available in the literature. Our experiments show that our method outperforms the competitors in the imputation of most of the features and on average.

To assess the possible impact of the proposed method in a concrete scenario, we provide a simple application of the imputed data in a survival classification task. We used a naïve Bayes (NB) classifier to distinguish between patients with long and short survival times by using only the information in their first three months of screening visits. Our results show that imputing the training set with the proposed method improves the prediction performance of the NB classifier on a hold-out test set, also achieving better performance than the classifier built on the training set imputed with the top competitor (MICE). By asserting the effectiveness of the proposed imputation method in enhancing the training data for a very simple classification algorithm with naïve hypotheses, we confirm its applicability in more complex and sophisticated analyses. Finally, we believe that the proposed methodology could be of great aid to clinicians since it enables the survival prediction of patients by employing only the information from their first three months of visits, regardless of possible missing values.

## Materials and methods

### Dataset

The dataset used in this work was extracted from the PARALS Register as follows. We selected the cohort of patients with first visit from January 1st, 2001 and follow-up up to July 18th, 2017, and excluded the ones having an onset that predated the first visit by five years or more (average ALS prognosis) in order to filter out clinical outliers. The selected cohort includes 700 patients, resulting in a dataset containing the information assessed over their subsequent screening visits, for a total of 6,726 visits.

The 25 variables collected in the dataset include some clinical features recorded during the first visit –the static ones– that are: patient sex, body-mass index (BMI) both premorbid and at diagnosis, a measure of respiratory functionality (forced vital capacity, FVC) at diagnosis, familiality of ALS, the result of a genetic screening over the most common ALS-associated genes, presence of frontotemporal dementia (FTD), site of disease onset (limb/bulbar), age at onset, diagnostic delay (time from ALS onset to diagnosis); the remaining features –the dynamic ones– are collected over visits and consist of: the presence/absence up to the current visit of non-invasive ventilation (NIV) and percutaneous endoscopic gastrostomy (PEG), that are two guideline-recommended interventions for symptom management in ALS, and the revised ALS Functional Rating Scale (ALSFRS-R) [47], which is a 12-item questionnaire rated on a 0–4 point scale evaluating the observable functional status and change for patients with ALS over time.

The time of the visit for each patient is expressed in months and set to zero in correspondence to the first visit, resulting in negative values for the onset delta. These variables are detailed in Table 1, according to their data type (continuous, ordinal, or categorical), with the percentage of native missing values and the static (S) or dynamic (D) nature of the feature. In this summary, for the NIV and PEG variables we reported the total number of patients who were administered these interventions.

**Table 1:** Dataset. For each feature, the type either static (S) or dynamic (D) is defined. For the continuous and ordinal features, percentage of native missing values and inter-quartile range (IQR) values at 25%, 50% and 75% are reported; for the categorical features, levels and corresponding percentage of instances are reported; for the NIV and PEG variables, we reported the total number of patients who were administered these interventions

Continuous features					Categorical features			
Feature	Type	% NA	IQR		Feature	Type	Levels	%
BMI premorbid [kg/m^2^]	S	2.08	23/25/28		sex	S	Female	47.6
BMI diagnosis [kg/m^2^]	S	0.91	22/24/27				Male	52.4
FVC diagnosis [%]	S	4.12	83/98/108				NA	0
age at onset [years]	S	0	56/64/70		familiality	S	No	91.4
diagnostic delay [months]	S	0	5/9/14				Yes	8.1
onset delta [months]	S	0	-18/-11/-6				NA	0.5
					genetics	S	C9orf72	7.1
							FUS	0.3
							SOD1	1.4
							TARDBP	1.6
Ordinal features							wild type	83.6
Feature	Type	% NA	IQR				NA	6.0
ALSFRS-R 1	D	0	2/3/4		FTD	S	No	53.0
ALSFRS-R 2	D	0	3/4/4				Yes	13.0
ALSFRS-R 3	D	0	2/3/4				NA	34.0
ALSFRS-R 4	D	0	2/3/4		onset site	S	Bulbar	34.4
ALSFRS-R 5	D	0	1/2/3				Limb	65.6
ALSFRS-R 6	D	0	1/2/3				NA	0
ALSFRS-R 7	D	0	1/3/3		NIV	D	No	59.6
ALSFRS-R 8	D	0	2/2/3				Yes	40.4
ALSFRS-R 9	D	0	0/1/3				NA	0
ALSFRS-R 10	D	0	3/4/4		PEG	D	No	31.9
ALSFRS-R 11	D	0	3/4/4				Yes	25.0
ALSFRS-R 12	D	0	4/4/4				NA	43.1

In order to develop and validate the imputation algorithms on independent data, we split the dataset in training (80% = 560 subjects, 5,507 visits) and test (20% = 140 subjects, 1,219 visits) sets, by stratifying the dataset over all variables.

### Imputation algorithm

In this work we developed a weighted k-NN approach to impute the missing values in the first three months of screening visits of each patient. We based our algorithm on the assumption that patients with similar characteristics share the same disease course over time. Patient similarity is assessed by using an apposite distance metric over their features.

Given a patient with a missing value to be imputed and a pool of other patients having that feature, the algorithm searches for the *k*-closest subjects in terms of disease progression similarity and infers the estimate for the missing value. First, the distance among the current patient and the other candidate subjects from the pool is computed. Then, a weighted average of the corresponding values in the *k* most similar patients is obtained and used as plausible estimate of the missing one. To impute the whole dataset, the procedure is iterated for each missing value of the given patient and then for each patient with missing values in their visits. The algorithm takes into account the temporal evolution of the data over visits and handles both the mixed nature of the data and the presence of missing values in the distance computation.

#### Adaptive k-NN sample construction

**Fig. 1:**
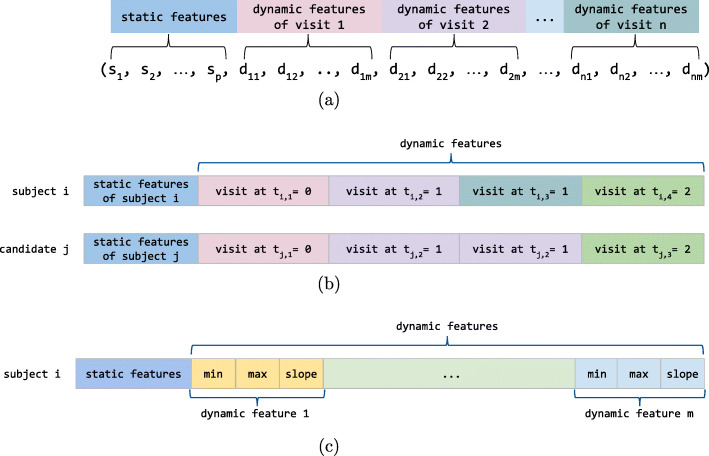
Sample construction for imputation and survival classification. **a** Sample construction for each patient with missing data to be imputed. **b** Candidate sample construction procedure. In this example, subject *i* has *n*=4 visits in the first three months of screening (one in the first month, two in the second and one in the third) while candidate *j* has 3 visits in this interval (one visit per month). Since the visit at *t*_*j*,2_ matches both visits at *t*_*i*,2_ and *t*_*i*,3_, its dynamic feature values are repeated twice in the resulting feature vector (sample). **c** Survival classification sample construction for each patient

To capture the temporal evolution of the features over subsequent visits, for a given patient *i* with missing data to be imputed, the algorithm builds a feature vector (k-NN sample) that contains the information recorded during his/her first three months of screening visits. The feature vector is created by binding the static information for that patient (constant throughout all his/her visits) to the dynamic ones in the [0,2] months time interval from the first visit in chronological order (with 0 being the first month). In our dataset, all the patients have between 1 and 4 visits in the first three months of screening: the algorithm adaptively builds k-NN samples whose length depends on the number of available visits for each subject to be imputed. Figure 1(a) illustrates the sample construction for subject *i*, with *p* being the number of static features, *m* the number of the dynamic ones, and *n* the number of his/her visits in the first three months of screening.

To identify the subjects in the pool of candidates having disease progression similar to subject *i*, the algorithm builds an analogous feature vector for each candidate neighbour with an available value in correspondence to the feature to be imputed. In more detail, each candidate neighbour *j* is temporally mapped over the current subject *i*, adaptively building a sample according to their matching time points. The feature vector of *j* is initialised with the subject’s static features. Let $\mathbf {t}_{i}=\left (t_{i,1}, t_{i,2}, \dots, t_{i,n}\right)$ be the time points of the visits in the first three months of screening for subject *i*. For each visit time point *t*_*i*,*l*_ of subject *i*, the closest-in-time visit of subject *j* within one month is selected. If no matching visit is found, candidate *j* is excluded from the k-NN search. Otherwise, the dynamic features of the matching visit are extracted and stacked to the feature vector of subject *j*; possible missing values in the matching visits of subject *j* are passed on his/her feature vector. Please notice that a candidate subject *j* may have repeated blocks of dynamic features in his/her feature vector corresponding to the same visit matching with multiple visits of subject *i*. Also notice that the feature vectors of the candidate subjects include the dynamic information of visits in the [0,3] months time interval from the first visit (that is, of the first four months of screening visits). Figure 1(b) schematically depicts the candidate sample construction procedure.

#### Weighted k-nearest neighbours

For a subject *i* with a missing value to be imputed, the wk-NN algorithm proceeds as follows. The features of the subject sample, together with his/her candidate samples, are normalised to the [0,1] interval in order to account for the difference among the ranges. Then, the distance between subject *i* and each candidate *j* is computed according to the following metric.

Let $\mathbf {v}=\left (v_{1}, v_{2}, \dots, v_{N}\right)$ and $\mathbf {u}=\left (u_{1}, u_{2}, \dots, u_{N}\right)$ be the feature vectors of, respectively, subject *i* and candidate *j*. Let *N*_stat_(**v**,**u**) and *N*_dyn_(**v**,**u**), be, respectively, the number of common non-missing static and dynamic features in **v** and **u**. Also, let *S*_categ_,*S*_ord_,*S*_cont_,*D*_categ_,*D*_ord_, and *D*_cont_ be the sets of indices of, respectively, the static categorical, the static ordinal, the static continuous, the dynamic categorical, the dynamic ordinal, and the dynamic continuous features in **v** and **u**. The distance between **v** and **u** is given by:

1 $$ {{}\begin{aligned} d(\mathbf{v}, \mathbf{u}) =& \frac{ n \cdot \left(\sum_{l \in S_{\text{categ}}} I(v_{l}, u_{l}) + \sum_{l \in S_{\text{ord}} \cup S_{\text{cont}}} |v_{l} - u_{l}| \right)}{n \cdot N_{\text{stat}}(\mathbf{v}, \mathbf{u}) + N_{\text{dyn}}(\mathbf{v}, \mathbf{u})} \\ & + \frac{\sum_{l \in D_{\text{categ}}} I(v_{l}, u_{l}) + \sum_{l \in D_{\text{ord}} \cup D_{\text{cont}}} |v_{l} - u_{l}| }{n \cdot N_{\text{stat}}(\mathbf{v}, \mathbf{u}) + N_{\text{dyn}}(\mathbf{v}, \mathbf{u})} \enspace, \end{aligned}}   $$

where *n* is the number of visits in the first three months of screening for subject *i* and *I*(*v*_*l*_,*u*_*l*_) is 0 if *v*_*l*_=*u*_*l*_ and 1 otherwise. If either *v*_*l*_ or *u*_*l*_, or both, are missing, the feature at index *l* does not contribute to the distance. The numerator is divided by the number of comparable features in *u* and *v* to normalise the distance on the number of common non-missing values. Because of the sample building procedure, each dynamic feature appears *n* times in the feature vectors: to re-balance the contribution of all the features to the similarity metrics, both the distance between static features and the count *N*_stat_(**v**,**u**) are multiplied by *n*.

At this point, a filtering step is performed: candidates with a number of comparable features with subject *i* smaller than the 90% of the total number of non-missing features in sample *i* (both computed with the same adjustment for the static features) are dropped.

Once the distances to all the candidates have been computed, the *k* nearest ones are selected and their values in correspondence to the feature to be imputed are used for the imputation: for continuous and ordinal features, after removing possible outliers (values outside 1.5 times the interquartile range above the upper quartile and below the lower quartile), the missing feature in *i* is imputed with the average of the selected values, each weighted by the inverse of the corresponding candidate distance; for categorical features, the missing feature in *i* is imputed with the mode of the selected values.

The procedure is repeated over all features with missing values in subject *i*. In our implementation, values previously imputed in *i* are not used for the subsequent imputations.

#### Weighted k-nearest neighbours with mutual information

We improved the wk-NN algorithm by including the cross-information among the features, given by the mutual information statistic, in the similarity metric (wk-NN MI). Unlike correlation metrics, the MI can measure the strength of both linear and nonlinear associations among features.

The MI among features is computed using the *infotheo* R package v1.2.0 [48]. For two discrete variables *X* and *Y* whose joint probability distribution is *p*_*XY*_(*x*,*y*)=*P*(*X*=*x*,*Y*=*y*), and marginal probability distributions are, respectively, *p*_*X*_(*x*)=*P*(*X*=*x*) and *p*_*Y*_(*y*)=*P*(*Y*=*y*), the mutual information between them, denoted MI(*X*,*Y*), is computed as:

2 $$ \text{MI}(X,Y)=\sum_{x\in \mathcal{X}} \sum_{y\in \mathcal{Y}} p_{XY}(x, y) \ln{\frac{p_{XY}(x, y)}{p_{X}(x)p_{Y}(y)}} \enspace.  $$

The marginal and joint probability distributions of *X* and *Y* are determined empirically from the data by a frequentist approach. Continuous variables (*X*) are discretised into $i=\sqrt [3]{N}$ intervals of equal width *w*=(max(*X*)− min(*X*))/*i*, where *N* is the number of samples of *X*.

Let *f* be the index of the feature currently being imputed in subject *i*, and let $\text {\bf {MI}}_{f}=\left (\text {MI}_{f,1}, \dots, \text {MI}_{f,f}, \dots,\text {MI}_{f,N} \right)$ be the MI values between the feature at index *f* and all the features in the sample. The MI values are then employed as weights for the distance computation in the wk-NN algorithm:

3 $$ {\begin{aligned} d_{f}(\mathbf{v}, \mathbf{u}) =& \frac{ n \cdot \left(\sum_{l \in S_{\text{categ}}} \text{MI}_{f,l} \cdot I(v_{l}, u_{l}) + \sum_{l \in S_{\text{ord}} \cup S_{\text{cont}}} \text{MI}_{f,l} \cdot |v_{l} - u_{l}| \right) }{n \cdot N_{\text{stat}}(\mathbf{v}, \mathbf{u}) + N_{\text{dyn}}(\mathbf{v}, \mathbf{u})} \\ & + \frac{ \sum_{l \in D_{\text{categ}}} \text{MI}_{f,l} \cdot I(v_{l}, u_{l}) + \sum_{l \in D_{\text{ord}} \cup D_{\text{cont}}} \text{MI}_{f,l} \cdot |v_{l} - u_{l}| }{n \cdot N_{\text{stat}}(\mathbf{v}, \mathbf{u}) + N_{\text{dyn}}(\mathbf{v}, \mathbf{u})}\enspace. \end{aligned}}   $$

**Fig. 2:**
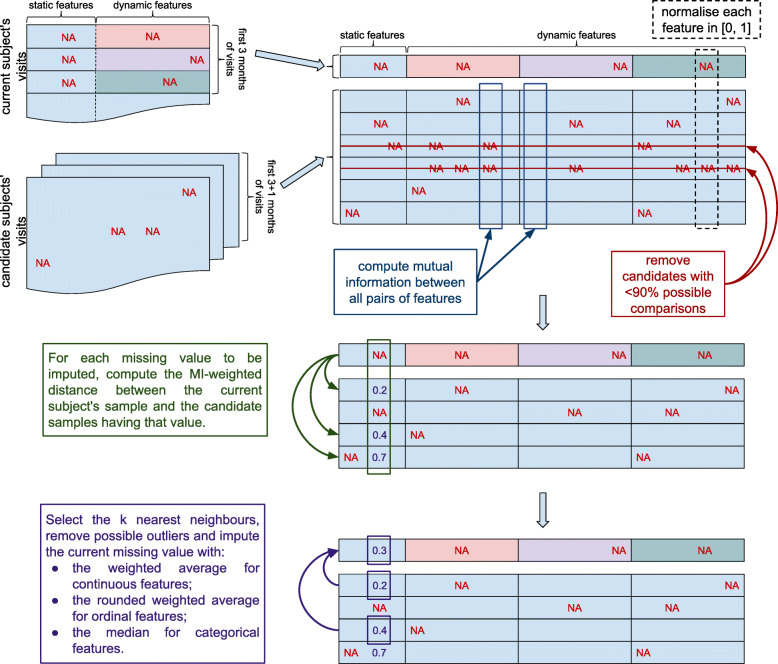
Algorithm workflow of the wk-NN MI imputation method


Please notice that here the distance among samples depends on the missing feature value currently being imputed, which means that the candidates chosen as nearest neighbours may change when imputing different features. An outline of the proposed imputation procedure is given in Fig. 2 and thoroughly described in Algorithm 1.

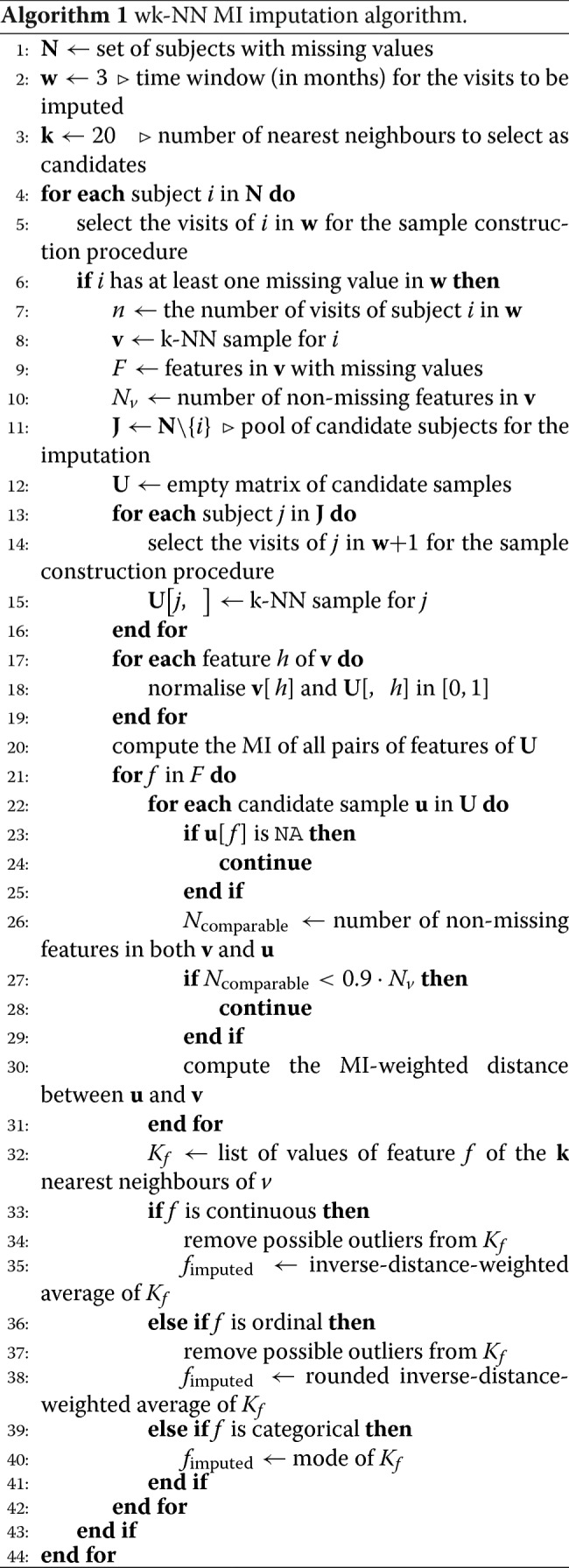


### Imputation performance metrics

To evaluate the performance of the developed imputation methods, we employed the normalised root-mean-square deviation (nRMSD) for the continuous and ordinal features and the proportion of falsely-classified (PFC) for the categorical ones. Let *f* be the index of a feature imputed in *T* patient visits: $\mathbf {v}_{f}^{\text {imp}}$ is the vector of imputed values for that feature and $\mathbf {v}_{f}^{\text {true}}$ is the vector of true measured values. If *f* is the index of a continuous or ordinal feature, the corresponding nRMSD is calculated over the *T* patient visits as:

4 $$ \text{nRMSD}_{f}= \frac{{\sqrt{ \frac{\sum_{i=1}^{T} \left(v_{i,f}^{\text{true}} - v_{i,f}^{\text{imp}} \right)^{2}}{T} }}}{\max(\mathbf{v}_{f}^{\text{true}}) - \min(\mathbf{v}_{f}^{\text{true}})}\enspace.  $$

Otherwise, if *f* is the index of a categorical feature, the corresponding PFC is calculated over the *T* patient visits as:

5 $$ \text{PFC}_{f} = \frac{\sum_{i=1}^{T} I(v_{i,f}^{\text{true}}, v_{i,f}^{\text{imp}}) }{T} \enspace,  $$

where $I(v_{i,f}^{\text {true}}, v_{i,f}^{\text {imp}})$ equals 0 if $v_{i,f}^{\text {true}} = v_{i,f}^{\text {imp}}$, and 1 otherwise.

In order to better analyse and compare the distribution of the error, we also computed the normalised absolute error (nAE) of each imputed continuous or ordinal value. The nAE for the imputed feature *f* of a given patient visit is given by:

6 $$ {\text{nAE}_{f}(i)=\frac{|v_{i,f}^{\text{true}} - v_{i,f}^{\text{imp}}|}{\max(\mathbf{v}_{f}^{\text{true}}) - \min(\mathbf{v}_{f}^{\text{true}})}} \enspace.  $$

Analysing the nAE distribution for each feature allows us to gain more insight on the quality of the imputation.

In all cases, the closer these metrics are to zero the better the imputation.

### Selecting the optimal number of nearest neighbours

The proposed wk-NN and wk-NN MI imputation methods require the user to select an adequate *k* (number of nearest neighbours) hyperparameter. This can be achieved by performing a cross validation scheme to test out different *k* values and select the best one. The patients in the dataset are partitioned into a user-defined number of folds. For a given *k* value, for each patient in a given fold, and for each feature, all the measured values corresponding to that feature are first removed at the same time from the patient’s visits, and then imputed by using all the subjects from the other folds as candidates.

By repeating this procedure for all folds, an imputed value is obtained for each known measurement, and the imputation quality for the current value of *k* can be assessed by using a chosen performance metric. This procedure can be repeated for several values of *k* in order to determine the best performing one to be finally used to impute the whole dataset. Moreover, by removing the values of only one feature at a time, the distribution and pattern of missing values in the dataset is generally preserved, which ensures the plausibility of the imputation performance results.

### Enhancing the performance of a survival classification task with data imputation

Patients with ALS exhibit a very high degree of variability in disease susceptibility and pathogenic mechanisms. This is one of the main reasons for the negative results of therapeutic trials conducted so far, as statistical variance masks treatment effects [49, 50]. An optimal trial design requires samples size estimation, which, in turn, requires some understanding of the natural progression of the disease. The accurate prediction of the survival time in ALS patients is of paramount importance, and could aid prognostic counselling, stratification of cohorts for pharmacological trials, and timing of interventions.

In order to evaluate the enhanced potential of the dataset imputed with the proposed method, we implemented a simple survival classification task. The PARALS register contains survival information for each patient, either in the form of date of death for the deceased ones or the date of the last visit for the censored ones. For each subject, we determined the survival outcome as the binary answer to the question “Does the subject survive for more than 3 years (36 months) from his/her first screening visit?”. The patients that were censored before the 36 months threshold were discarded since we were unable to answer the question. The number of patients in the training set was thus reduced to 545 (from the initial 560), and the number of patients in the test set was reduced to 138 (from the initial 140). The 36 months threshold was selected because it splits the patients into two almost equal sets.

For each patient, we built a *survival sample* – a feature vector able to encode the disease progression in his/her first three months of visits, as follows. For each dynamic feature in this time range, we computed three derived features, namely the minimum, maximum, and the slope. The slope was obtained by fitting a linear regression model on the temporal series constituted by the values of the feature collected over the three months interval. These values were then used together with the static features to construct a fixed-length vector (53 features in total) used as an input sample for our classification task (see Fig. 1(c)). The survival samples constructed on the original data (that is, before imputation) carry over their missing values. When handling missing static features, the missing values were simply carried over to the constructed samples. In case of missing dynamic features, missing values are reported in the corresponding derived features that could not be computed due to data missingness.

For this classification task we employed the naïve Bayes classifier [51] implemented in the *e1071* R package v1.7-2 [52].

#### Naïve bayes models

Naïve Bayes is a simple learning algorithm that utilises Bayes’ theorem in conjunction with the “naïve” assumption that, given the class label, every pair of features is conditionally independent. A NB classifier considers the contribution of each feature to the given class probability as independent, regardless of possible correlations. Although this assumption is often violated in practice, NB classifiers often achieve competitive classification results [53]. Because of theirs computational efficiency and many other desirable features, NB classifiers are widely used in practice. A brief introduction to the method is reported in Additional file 1.

In order to evaluate the effect of the different imputation techniques on the classification task, and to further assess the performance of the proposed algorithm, we trained five NB models on five distinct sets of survival samples. First, starting from the original non-imputed training set composed of the first three months of patient visits, we built the corresponding training set of survival samples with their native missing values, from here on referred to as *original dataset*. From this first set we obtained two other sets for the complete case analysis: the *complete cases dataset* obtained by selecting only the survival samples without missing values, resulting in 252 survival samples, and the *complete features dataset* obtained by selecting only the features without missing values, resulting in 44 remaining features in the survival samples. Finally, we built two other training sets of survival samples for the classification task by imputing the first three months of patient visits from the training set once with the proposed algorithm (wk-NN MI) and once with the best performing competitor.

The models were used to predict the set of test samples obtained from the non-imputed first three months of patient visits in the original test set.

## Results and discussion

### Comparison with the other imputation methods

We compared the proposed algorithm with the three state-of-the-art imputation methods, namely Amelia II (*Amelia* R package v1.7.5), missForest (*missForest* R package v1.4) and MICE (*mice* R package v3.6.0). We also introduced a random version of our algorithm, k-random neighbours (k-RN), that randomly samples a subset of *k* subjects from the pool of available candidates, to be used as a baseline for the imputation performance assessment. The selection of the optimal hyperparameter values for all the employed imputation methods is reported in Additional file 1.

#### Performance comparison on the training set

On the training set, the imputation performance was evaluated with the LOOCV setting described earlier: for each subject, all the measured values of his/her features were removed one feature at a time, and were then imputed using the competitor methods. The imputed values obtained by each method were compared to the true ones, and the average error was evaluated for each feature.

Tables 2, 3 and 4 show the average error (in terms of nRMSD or PFC) obtained on the training set for each continuous, ordinal and categorical feature, respectively. The proposed wk-NN MI imputation method outperforms the competitors on average and on the majority of the features. For the continuous features, the average nRMSD score obtained by wk-NN MI with the optimal *k*=20 is 0.1195 against 0.1539 of wk-NN with the optimal *k*=10, 0.1651 of Amelia II, 0.1572 of MICE, and 0.1784 of missForest. For the ordinal features, the average nRMSD score obtained by wk-NN MI is 0.1182 against 0.1550 of wk-NN, 0.1751 of Amelia II, 0.1521 of MICE, and 0.1728 of missForest. For the categorical features, the average PFC score obtained by wk-NN MI is 0.1198 against 0.1323 of wk-NN, 0.2589 of Amelia II, 0.1761 of MICE, and 0.1900 of missForest. In the three tables, we also report the performances for the k-RN baseline, computed for *k*=10 and *k*=20: the obtained performances outperform the baseline.

**Table 2:** nRMSD scores for the continuous features in the training set. The best performances are highlighted in bold

Features	Imputation methods						
	Amelia II	MICE	missForest	k-RN	wk-NN	k-RN	wk-NN MI
				*k*=10	*k*=10	*k*=20	*k*=20
BMI premorbid	0.1012	0.0960	0.1323	0.1634	0.1286	0.1617	**0.0731**
BMI diagnosis	0.1560	0.1069	0.1476	0.1750	0.1457	0.1687	**0.0965**
FVC diagnosis	0.2466	0.2463	0.2534	0.1970	0.1876	0.1953	**0.1839**
age at onset	0.2355	0.2362	0.2393	0.1855	0.1748	0.1820	**0.1735**
diagnostic delay	0.1150	0.1218	0.1316	0.1484	0.1282	0.1495	**0.0850**
onset delta	0.1362	0.1362	0.1665	0.1848	0.1584	0.1778	**0.1049**
Average	0.1651	0.1572	0.1784	0.1757	0.1539	0.1725	**0.1195**

**Table 3:** nRMSD scores for the ordinal features in the training set. The best performances are highlighted in bold

Features	Imputation methods						
	Amelia II	MICE	missForest	k-RN	wk-NN	k-RN	wk-NN MI
				*k*=10	*k*=10	*k*=20	*k*=20
ALSFRS-R 1	0.1959	0.1540	0.1788	0.2454	0.1529	0.2390	**0.1249**
ALSFRS-R 2	0.1644	0.1433	0.1684	0.1904	0.1394	0.1907	**0.1218**
ALSFRS-R 3	0.1768	0.1387	0.1679	0.2175	0.1331	0.2130	**0.1133**
ALSFRS-R 4	0.2173	0.1916	0.2145	0.2516	0.1606	0.2455	**0.1472**
ALSFRS-R 5	0.2183	0.1863	0.2179	0.2812	0.1763	0.2727	**0.1394**
ALSFRS-R 6	0.2064	0.2015	0.2113	0.2864	0.1849	0.2773	**0.1513**
ALSFRS-R 7	0.1953	0.1696	0.1833	0.2645	0.1544	0.2550	**0.1295**
ALSFRS-R 8	0.2021	0.1488	0.1651	0.2460	0.1470	0.2377	**0.1138**
ALSFRS-R 9	0.2655	0.2405	0.2268	0.3744	0.2222	0.3657	**0.1589**
ALSFRS-R 10	0.1060	0.1093	0.1565	0.2523	0.1668	0.2475	**0.0943**
ALSFRS-R 11	0.0854	0.0982	0.1340	0.2446	0.1585	0.2403	**0.0847**
ALSFRS-R 12	0.0682	0.0434	0.0485	0.0933	0.0637	0.0908	**0.0391**
Average	0.1751	0.1521	0.1728	0.2457	0.1550	0.2396	**0.1182**

**Table 4:** PFC scores for the categorical features in the training set. The best performances are highlighted in bold

Features	Imputation methods						
	Amelia II	MICE	missForest	k-RN	wk-NN	k-RN	wk-NN MI
				*k*=10	*k*=10	*k*=20	*k*=20
sex	0.4859	0.4416	0.4463	0.5160	0.3974	0.4831	**0.3823**
familiality	0.1646	0.1268	0.1372	0.0842	0.0823	0.0842	**0.0738**
genetics	0.3310	0.1781	0.1751	0.0956	0.0895	0.0956	**0.0815**
FTD	0.3295	0.2642	0.3565	0.2060	0.2003	0.1960	**0.1903**
onset site	0.2957	0.1516	0.1403	0.3672	0.1017	0.3484	**0.0800**
NIV	0.1111	0.0556	0.0537	0.0518	0.0480	0.0518	**0.0235**
PEG	0.0948	0.0150	0.0208	**0.0069**	**0.0069**	**0.0069**	**0.0069**
Average	0.2589	0.1761	0.1900	0.1897	0.1323	0.1809	**0.1198**

**Fig. 3:**
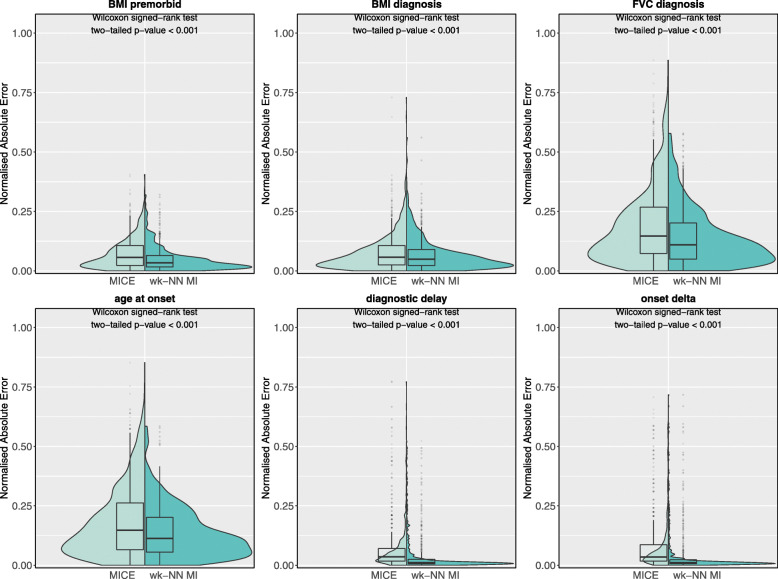
Normalised absolute error distributions obtained with MICE and wk-NN MI (with *k*=20) on the continuous features of the training set

To verify that the performance improvement was in fact statistically significant, we analysed the nAE distributions and PFC values obtained by wk-NN MI and MICE (the best performing among the competitor methods) on, respectively, the continuous/ordinal and categorical features. Figure 3 shows the nAE distributions obtained on the training set for the continuous features. The plots show that wk-NN MI yields lower nAE values in all features. We also performed two-tailed Wilcoxon signed-rank tests [54] to assess the difference between the distributions: the obtained p-values are all smaller than 0.001, confirming that the difference is statistically significant. The Wilcoxon signed-rank test is a non-parametric statistical test used to assess whether the population mean ranks differ in a paired samples setting. This test can be used to determine whether two paired samples were selected from populations having the same distribution. We employed this non-parametric test to asses whether there is any statistically significant difference between the nAE distributions (which are very skewed and cannot be assumed to be normally distributed) obtained on continuous and ordinal data by different imputation methods.

**Fig. 4:**
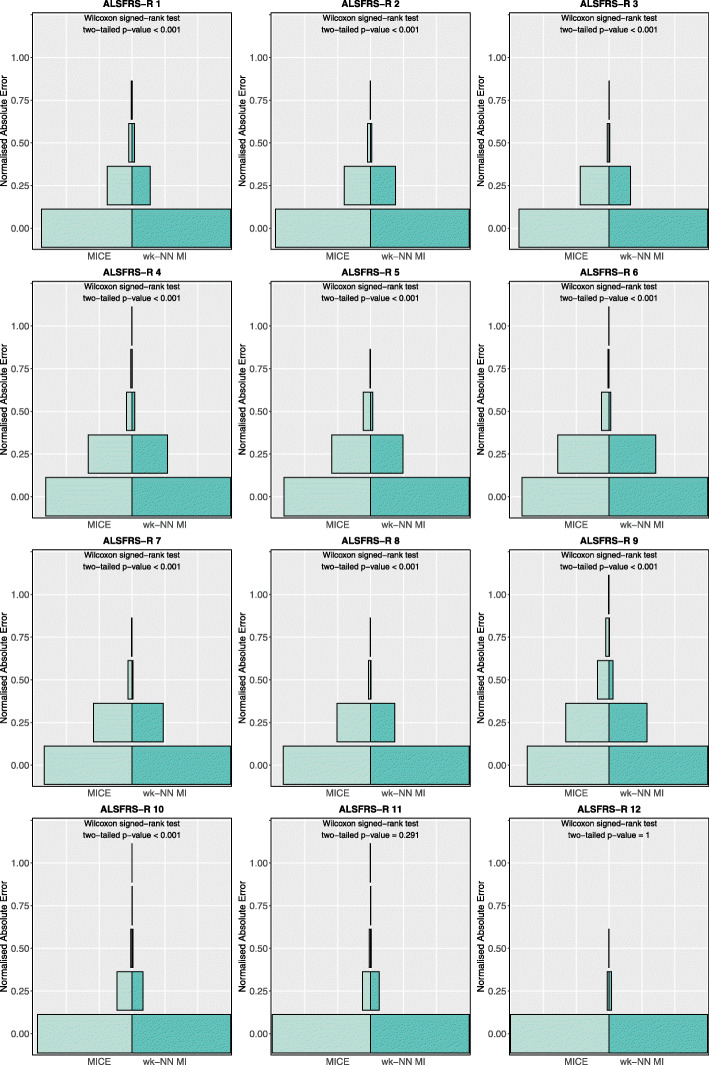
Normalised absolute error distributions obtained with MICE and wk-NN MI (with *k*=20) on the ordinal features of the training set

Figure 4 shows the nAE distributions obtained on the training set for the ordinal features. The plots show that wk-NN MI yields lower nAE values on 10 out of 12 features (ALSFRS-R scores 1 to 10). We also performed two-tailed Wilcoxon signed-rank tests with Pratt’s correction (since the nAE values on the ALSFRS-R variables can only assume values in {0,0.25,0.5,0.75,1}, the signed-rank test has many “ties”) to assess the difference between the distributions: the obtained p-values are smaller than 0.001 for the *ALSFRS-R* scores 1 to 10 which confirms that the difference is statistically significant for these features. Lastly, the tests showed that for *ALSFRS-R* 11 and 12 there was no statistically significant difference between wk-NN MI and MICE.

**Fig. 5:**
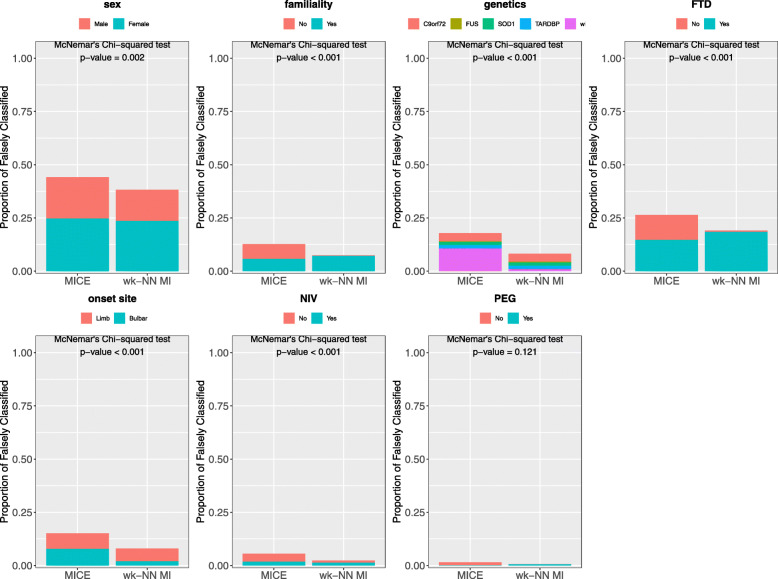
Proportion of falsely classified obtained with MICE and wk-NN MI (with *k*=20) on the categorical features of the training set

Figure 5 compares the PFC values obtained by wk-NN MI and MICE. The plots show that wk-NN MI outperforms MICE in all the categorical features, resulting in a significant difference in 6 out of 7 of them, namely in *sex*, *familiality*, *genetics*, *FTD*, *onset site*, and *NIV*, while showing no significant improvement for *PEG*. We also performed McNemar’s Chi-squared test [55] which confirmed that the difference is statistically significant in these 6 features. McNemar’s Chi-squared test is a statistical test used on paired categorical data. It is applied to 2×2 dichotomous contingency tables with paired samples, to determine whether there is “marginal homogeneity”, that is, the row and column marginal frequencies are equal. When comparing two classifiers, each sample can be either be classified correctly or miss-classified by each classifier, and thus a 2×2 dichotomous contingency table can be built. The null hypothesis of “marginal homogeneity” would mean there is no difference between the two classifiers. The imputation of categorical data can be seen as a classification task, and thus, McNemar’s Chi-squared test can be used to determine if the difference between two imputation methods is statistically significant.

#### Performance comparison on the test set

After selecting the methods’ hyperparameters on the training set, we compared the performance of the proposed imputation method against the competitors on the test set. For each patient in the test set, we removed all the known measurements from his/her visits, one feature at a time, and imputed the missing values by using all the training set subjects as candidates. This setting represents the common situation where new subjects are continuously added to an existing dataset of clinical records and some of their values are natively missing. For Amelia II, MICE and missForest, we bound the records of the first three months of visits for the given patient in the test set with all the information on the training set in a single data frame, which was then used as an input for these imputation algorithms. Finally, we compared the imputed values obtained by each method with the true ones.

The imputation results on the test set are shown in Tables 5, 6 and 7 for each continuous, ordinal and categorical feature, respectively. Results on the held-back test set confirm that the proposed wk-NN MI imputation method outperforms the competitors on average and on the majority of the features. For the continuous features, the average nRMSD score obtained by wk-NN MI is 0.1332 against 0.1624 of wk-NN, 0.1803 of Amelia II, 0.1731 of MICE, and 0.2011 of missForest. For the ordinal features, the average nRMSD score obtained by wk-NN MI is 0.1274 against 0.1561 of wk-NN, 0.2654 of Amelia II, 0.1542 of MICE, and 0.1740 of missForest. For the categorical features, the average PFC score obtained by wk-NN MI is 0.1303 against 0.1456 of wk-NN, 0.2646 of Amelia II, 0.1900 of MICE, and 0.1966 of missForest. The baseline was also outperformed by the proposed wk-NN approaches.

**Table 5:** nRMSD scores for the continuous features in the test set. The best performances are highlighted in bold

Features	Imputation methods						
	Amelia II	MICE	missForest	k-RN	wk-NN	k-RN	wk-NN MI
				*k*=10	*k*=10	*k*=20	*k*=20
BMI premorbid	0.1302	0.1353	0.1787	0.2047	0.1692	0.2034	**0.1105**
BMI diagnosis	0.1459	0.1227	0.1653	0.1968	0.1665	0.2033	**0.1145**
FVC diagnosis	0.2481	0.2401	0.2584	0.2036	0.1821	0.1980	**0.1752**
age at onset	0.2799	0.2650	0.2781	0.2024	0.1847	0.2061	**0.1823**
diagnostic delay	0.1286	0.1228	0.1350	0.1481	0.1209	0.1422	**0.0958**
onset delta	0.1489	0.1529	0.1910	0.1785	0.1512	0.1686	**0.1210**
Average	0.1803	0.1731	0.2011	0.1890	0.1624	0.1869	**0.1332**

**Table 6:** nRMSD scores for the ordinal features in the test set. The best performances are highlighted in bold

Features	Imputation methods						
	Amelia II	MICE	missForest	k-RN	wk-NN	k-RN	wk-NN MI
				*k*=10	*k*=10	*k*=20	*k*=20
ALSFRS-R 1	0.3148	0.1852	0.1852	0.2467	0.1609	0.2528	**0.1457**
ALSFRS-R 2	0.2680	0.1852	0.2122	0.2197	0.1527	0.2049	**0.1424**
ALSFRS-R 3	0.2663	0.1673	0.1504	0.2443	0.1504	0.2265	**0.1416**
ALSFRS-R 4	0.2832	0.1913	0.1852	0.2770	0.1813	0.2762	**0.1602**
ALSFRS-R 5	0.3012	0.1741	0.2060	0.3039	0.1714	0.2873	**0.1496**
ALSFRS-R 6	0.3035	0.1768	0.1973	0.3141	0.1701	0.2996	**0.1631**
ALSFRS-R 7	0.2873	0.1687	0.1800	0.2762	0.1550	0.2787	**0.1416**
ALSFRS-R 8	0.2910	0.1550	0.1550	0.2645	0.1519	0.2514	**0.1153**
ALSFRS-R 9	0.3189	0.2192	0.2774	0.3709	0.2491	0.3549	**0.1800**
ALSFRS-R 10	0.1845	0.0903	0.1481	0.2410	0.1416	0.2462	**0.0648**
ALSFRS-R 11	0.1938	0.0941	0.1408	0.2316	0.1340	0.2415	**0.0716**
ALSFRS-R 12	0.1728	**0.0432**	0.0506	0.1013	0.0551	0.0990	0.0529
Average	0.2654	0.1542	0.1740	0.2576	0.1561	0.2516	**0.1274**

**Table 7:** PFC scores for the categorical features in the test set. The best performances are highlighted in bold

Features	Imputation methods						
	Amelia II	MICE	missForest	k-RN	wk-NN	k-RN	wk-NN MI
				*k*=10	*k*=10	*k*=20	*k*=20
sex	0.4440	0.4813	0.4366	0.5560	0.4366	0.4440	**0.3955**
familiality	0.2724	0.0970	0.1381	**0.0597**	**0.0597**	**0.0597**	0.0821
genetics	0.3166	0.2124	0.1776	0.1506	0.1506	0.1506	**0.1351**
FTD	0.4749	0.3575	0.3911	**0.2179**	0.2626	0.2235	0.2346
onset site	0.2910	0.1418	0.1343	0.4552	0.0896	0.4664	**0.0522**
NIV	0.0485	0.0299	0.0634	0.0410	0.0149	0.0410	**0.0075**
PEG	**0.0050**	0.0101	0.0352	**0.0050**	**0.0050**	**0.0050**	**0.0050**
Average	0.2646	0.1900	0.1966	0.2122	0.1456	0.1986	**0.1303**

**Fig. 6:**
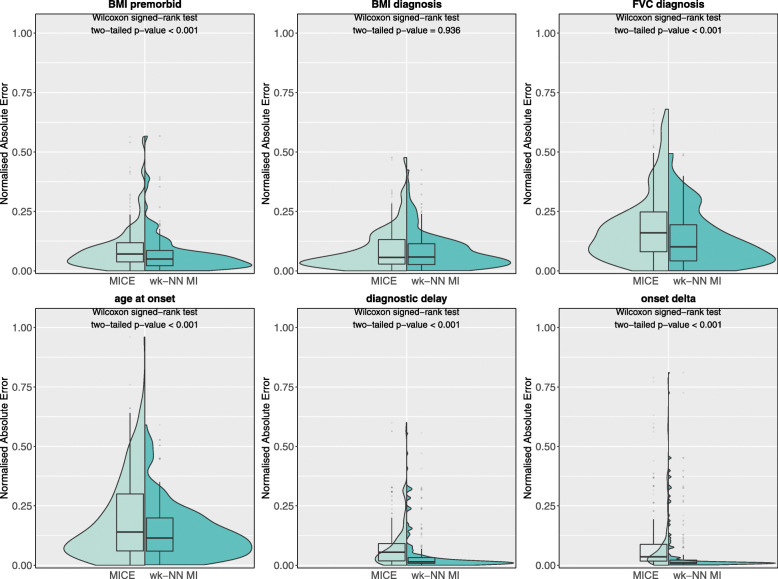
Normalised absolute error distributions obtained with MICE and wk-NN MI (with *k*=20) on the continuous features of the test set

We also analysed the nAE distributions and PFC values obtained by wk-NN MI and MICE (the best performing among the competitor methods) on, respectively, the continuous/ordinal and categorical features. Figure 6 shows the nAE distributions obtained on the test set for the continuous features. The plots and the two-tailed Wilcoxon signed-rank tests show that wk-NN MI yields statistically significant lower nAE values in 5 out of 6 features, namely *BMI premorbid*, *FVC diagnosis*, *age at onset*, *diagnostic delay*, and *onset delta*. The two methods did not obtain statistically significant differences in the imputation of *BMI diagnosis*.

**Fig. 7:**
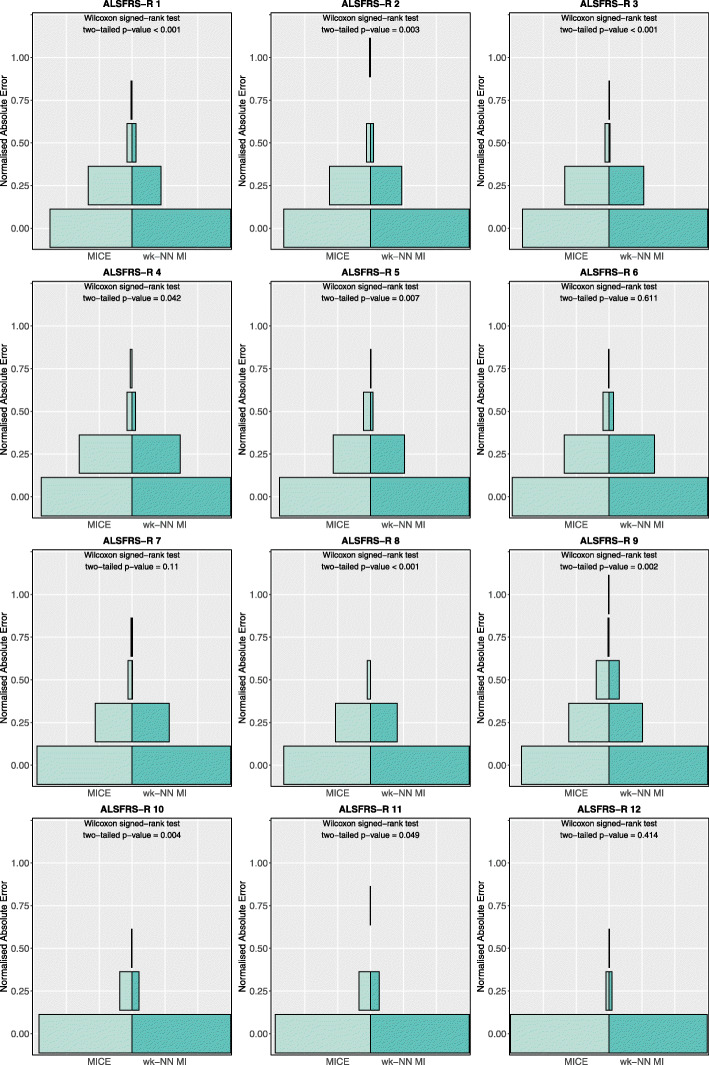
Normalised absolute error distributions obtained with MICE and wk-NN MI (with *k*=20) on the ordinal features of the test set

Figure 7 shows the nAE distributions obtained on the test set for the ordinal features. The plots and the two-tailed Wilcoxon signed-rank tests with Pratt’s correction show that wk-NN MI yields statistically significant lower nAE values on 9 out of 12 features (*ALSFRS-R* scores 1 to 5 and 8 to 11) at the 0.05 level. Lastly, the tests showed that for *ALSFRS-R* 6, 7 and 12 there was no statistically significant difference between wk-NN MI and MICE.

**Fig. 8:**
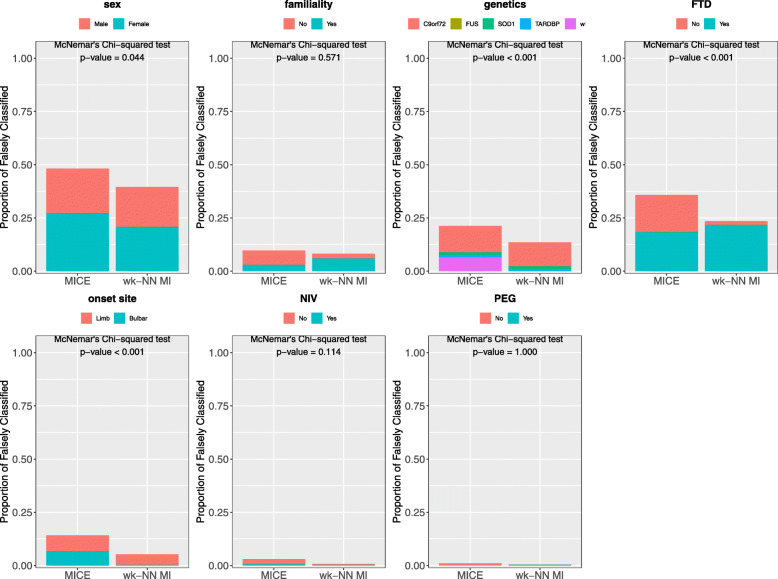
Proportion of falsely classified obtained with MICE and wk-NN MI (with *k*=20) on the categorical features of the test set

Figure 8 compares the PFC values obtained by wk-NN MI and MICE. The plots and the McNemar’s Chi-squared tests show that wk-NN MI outperforms MICE in 4 out of 7 categorical features, namely in *sex*, *genetics*, *FTD*, and *onset site*, at the 0.05 statistical significance level. No statistically significant improvements are obtained for *familiality*, *NIV* and *PEG*.

### Survival classification results

**Fig. 9:**
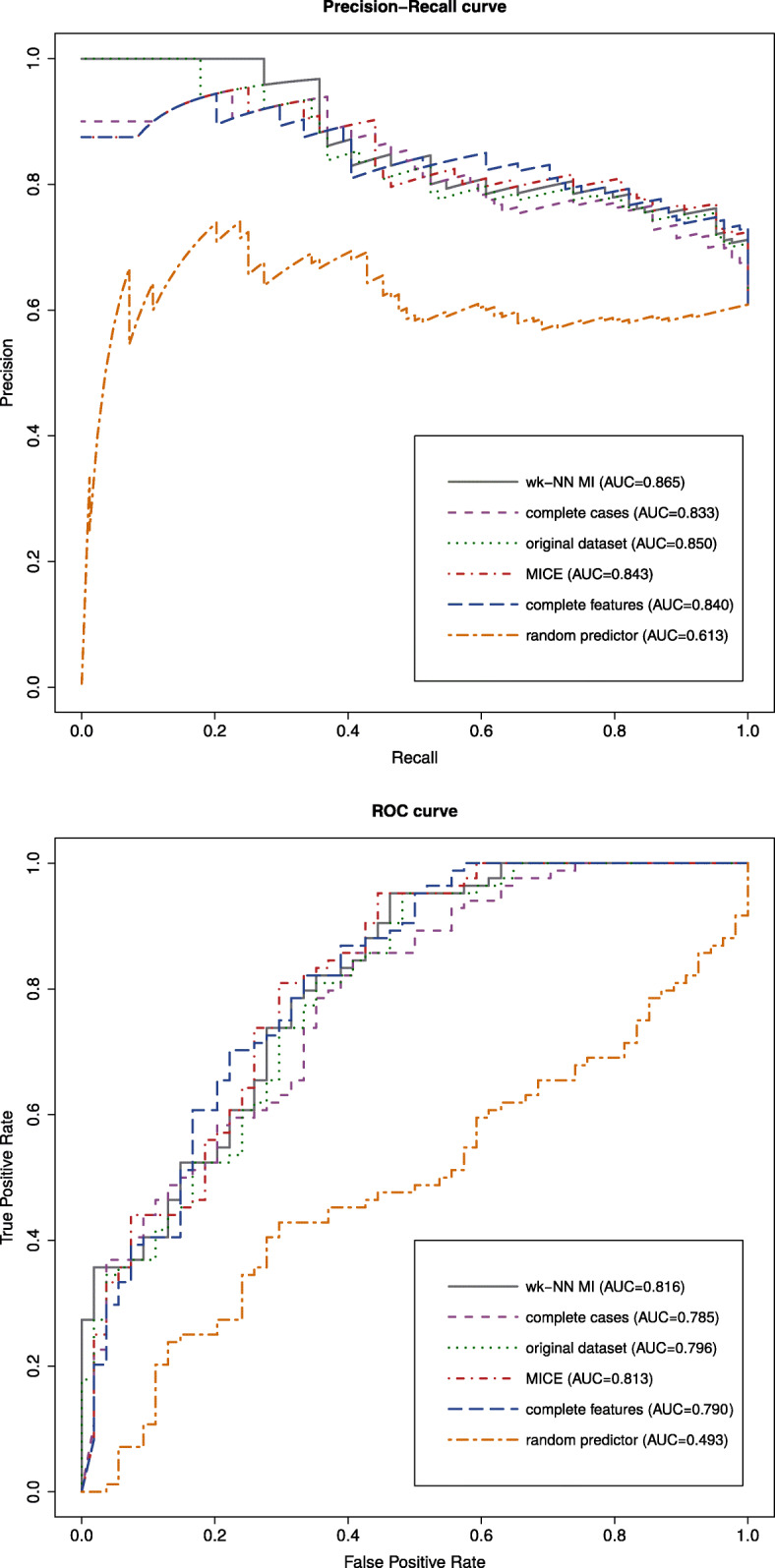
Precision-Recall and ROC plots of the naïve Bayes classifiers. The plots show that the imputation of the training set with the proposed method improves the classification performance of a naïve Bayes classifier

In this section we report the results of the survival classification procedure. Figure 9 gives the Precision-Recall (PR) and Receiver Operating Characteristic (ROC) plots of the NB classifiers trained on the five different sets of training samples. These plots were obtained by thresholding on the class label probabilities obtained by the NB classifiers for each survival sample. We also included the PR and ROC plots of a random predictor as a baseline. To ensure that the performance improvement is statistically significant, we computed the absolute classification error of the NB classifiers for each classification sample in the test set. The absolute classification error of each sample was computed as the absolute value of the difference between the class label and the predicted class probability. We performed two-tailed Wilcoxon signed-rank tests to assess the difference between the errors.

As a first result, we observe that the proposed method improves the prediction capabilities of a NB classifier: indeed, the PR curve achieves a perfect precision score of 1.0 for wider recall values. Moreover, the proposed method obtains the highest Area Under the Curve (AUC) value of 0.865. The improvement is somewhat less noticeable in terms of ROC curves and ROC-AUCs, although we can see that the proposed method improves the false positive rate which stays at zero for a wider true positive rate interval. The statistical test on the absolute classification error compared to all the other classifiers obtained p-values smaller than 0.001, confirming that the improvement is statistically significant.

Interestingly enough, the complete cases (PR-AUC=0.833 and ROC-AUC=0.785) and complete features analyses (PR-AUC=0.840 and ROC-AUC=0.790) worsen the prediction quality of the classifier with respect to the original dataset (PR-AUC=0.850 and ROC-AUC=0.796). The two-tailed Wilcoxon signed-rank tests’ p-value when comparing the complete cases and complete features analyses with the original dataset are <0.001 and 0.022, respectively, while there is no statistically significant difference between the complete cases and the complete features analyses (p-value =0.379). The loss of information resulting from simply ignoring samples or entire columns with missing data hinders the precision of the classifier. On the other hand, the NB classifier can effectively learn from the survival samples with their native missing values, as reflected by the prediction results.

By comparing the predictions of the NB classifier trained on the original dataset (PR-AUC=0.850 and ROC-AUC=0.796) with the ones trained on the two imputed datasets, we can see how the imputation quality can affect the classification performance: the performance improves when the patient data are imputed with wk-NN MI (PR-AUC=0.865 and ROC-AUC=0.816), while it worsens when using the best competitor for the imputation (MICE), as can be seen from its PR and ROC curves which do not achieve a perfect precision of 1 or a perfect false positive rate of 0 for any interval of recall/true positive rate.

## Conclusions

In this work we developed a weighted k-NN-based imputation approach, able to plausibly fill in the missing values in an ALS disease register. The best performing method, the proposed weighted k-NN with MI with *k*=20, outperforms the state-of-the-art algorithms in terms of imputation accuracy, on continuous, ordinal and categorical variables.

The advantages of the proposed approach are manifold. While many imputation methods require stringent assumptions on the nature of the missing data, a k-NN-based imputation only requires the presence of some relationship between the variable with the missing value and the other variables. The imputed values are always in the dynamic range of the existing data. Furthermore, the selection of a small *k* parameter ensures a good compromise between performance and the need to preserve the original distribution of the data, a very important characteristic any imputation method should satisfy.

The proposed method employs the MI values between feature pairs as weights in the distance computation of the wk-NN procedure. The results show that wk-NN MI outperforms the wk-NN approach, confirming that the MI can be effectively used to exploit the cross-information of the features for the imputation task.

We showed that the proposed algorithm is able to handle mixed-type data effectively, that is, patient records composed of categorical, ordinal and continuous features, each of which can be either static or dynamic, and with different distributions. In our method, thanks to the sample construction procedure described in *Adaptive k-NN Sample Construction*, the temporal evolution of the data over subsequent visits is captured and exploited for the imputation. Furthermore, our method does not require a dataset of complete cases to perform the imputation because of the distance metric used. We only used information from the training set to impute the subjects of the test set in order to simulate the real-world scenario where new subjects populate the disease register a few at a time.

Finally, we provided a simple survival classification task as a potential application example of the proposed imputation method. Our results show that the imputation of the missing values in the training dataset improves the predictions of a Naïve Bayes classifier. Since the NB represents a very simple classification technique, we believe that more complex and sophisticated analyses could also benefit from our imputation method.

For all these reasons, we believe that our method is potentially applicable in diverse contexts where imputation is needed. The final aim of this work is to provide a tool that can enhance the quality and the quantity of the data employed in analytics tasks, to improve and accelerate translational research. Concretely, the tool will allow clinicians to effectively use the information collected in a limited time interval by curing the possible presence of missing data.

The specific employment of the method in the context of epidemiological ALS registers will enable the development and application of machine learning and data mining methods for the prediction of ALS disease prognosis, as well as the identification of related biomarkers. As novel clinical registers covering wider patient populations and new clinical variables (for instance, new genetic test results, different functional scale measures) will become available, missing values arising from the aggregation with older datasets could be imputed with the proposed approach. We also believe that the proposed methodology could be of great aid in other disease registers containing static and dynamic mixed-type data as well.

The proposed algorithm is able to impute missing data in a fixed time window (that is, the first three months of patients’ visits). We plan to extend its imputation capabilities to the whole patients’ visits history with a sliding-window approach. Moreover, other distance metrics with more sophisticated weighting schemes could yield better imputation results. We will investigate these issues in our future work.

## Data Availability

The datasets generated and/or analysed during the current study are not publicly available in order to ensure the patients’ rights to privacy and anonymity and to prevent inappropriate secondary analyses. The proposed algorithm was implemented in the *wkNNMI* R package and is freely available from CRAN at https://cran.r-project.org/package=wkNNMI.

## Abbreviations

1-NN: 1-Nearest Neighbours
ALS: Amyotrophic Lateral Sclerosis
ALSFRS-R: Revised ALS Functional Rating Scale
AUC: Area Under the Curve
BMI: Body-mass index
DREAM: Dialogue for Reverse Engineering Assessments and Methods
FVC: Forced Vital Capacity
FTD: Frontotemporal Dementia
GP: Gaussian Process
k-NN: k-Nearest Neighbours
k-RN: k-Random Neighbours
LOOCV: Leave-One-Out Cross Validation
MAR: Missing At Random
MCAR: Missing Completely At Random
MI: Mutual Information
MICE: Multivariate Imputation by Chained Equations
ML: Machine Learning
MNAR: Missing Not At Random
MVN: Multivariate Normally Distributed
nAE: Normalised Absolute Error
NB: Naïve Bayes
NIV: Non-Invasive Ventilation
NN: Nearest Neighbours
nRMSD: Normalised Root-Mean-Square Deviation
PARALS: Piemonte and Valle d’Aosta Register for ALS
PEG: Percutaneous Endoscopic Gastrostomy
PFC: Proportion of Falsely Classified
PF: Precision-Recall
ROC: Receiver Operating Characteristic
wk-NN MI: Mutual Information-weighted k-Nearest Neighbours
wk-NN: weighted k-Nearest Neighbours
